# The World Allergy Organization Today

**DOI:** 10.1097/WOX.0b013e31817dc539

**Published:** 2008-06-15

**Authors:** G Walter Canonica

**Affiliations:** 1 President, World Allergy Organization

## 

Does the world really need a global allergy organization? As the individual honored to be president of the World Allergy Organization (WAO) at this exciting time in the organization's history, you would not expect my reply to be anything other than a resounding YES! But the undeniable fact is that allergy continues as a growing problem in most areas of the world, and a major global problem requires a coordinated global response.

The WAO started life more than 50 years ago as the International Association of Allergology and Clinical Immunology (IAACI). The IAACI organized triennial congresses to bring together the relatively small number of allergists, clinical immunologists, and organ-based specialists with a particular interest in allergic diseases. As the global burden of allergy increased, it became clear that an organization with a far wider scope of activity was needed to address the many issues surrounding this burgeoning clinical problem. In 1998, a decision was taken by the new Board of Directors of IAACI to change the name to World Allergy Organization, as the organization moved to adopt a greatly expanded role.

What are the major issues that WAO addresses? And why is WAO the right organization for the job? First, we are a federation of national allergy and clinical immunology societies and regional allergy organizations. Our members range from societies with several thousands of members and complex management structures, to small societies of tens of members, coordinated solely by the efforts of volunteers. Our larger and economically stronger member societies have developed the infrastructure to promote the specialty of allergy at a national level, to develop treatment guidelines and recommendations, and to train new generations of allergists; they have vast educational resources and expertise to share. Our smaller member societies welcome the opportunity to benefit from the pool of expertise available to help them develop the specialty of allergy at a national level; the role of WAO here is to distill the available resources and to act as an interface for transfer of information and knowledge between member societies.

Second, the WAO has the role of promoting knowledge about allergic diseases through education appropriately targeted for all physicians who see patients with allergy, in the pragmatic recognition that the vast majority of patients with allergy may never see a trained allergist. Thus, we are expanding our educational programs for the undergraduate, for the primary care physician, and the organ-based specialist, as well as the allergists who form our core membership. Our congresses now take place every 2 years, and registrations continue to grow--a further evidence of the need for a global organization to coordinate international collaboration between clinicians and scientists.

Third, the WAO has the responsibility of promoting the specialist skills of allergists to ensure recognition of the specialty. Although we share the care of our patients with allergy with other clinicians, the allergist is the best port of call for the atopic patient with single or multiple allergic symptoms, and optimal care for the patient is clearly in the best clinical interests of patients and the economic interests of society. Even a small core of trained allergists and interested organ-based specialists working together in each country can make a difference to how patients are treated. The development of new allergy societies through the Emerging Societies Program, in collaboration with the American College of Allergy, Asthma, and Immunology, has been a very rewarding and exciting area of our work. New WAO Special Committees will address some of the unique issues that require the skill sets and special knowledge of the allergist--Drug Allergy, Anaphylaxis, Immunotherapy, the Effect of Climate Change on Allergy, Clinical Trials in Allergy and Immunology, Food Allergy, Allergy Diagnosis. No other global organization is better placed to consider, advise upon, and educate the world about these important topics.

The fourth role of WAO is to act as the global voice of allergy. We represent allergy at the World Health Organization, and our collaboration with the World Health Organization, in particular through the Global Alliance against Chronic Respiratory Disease (GARD) program, is another area where a global voice for allergists adds particular value. Our research council is working with GARD to identify gaps in our knowledge about the epidemiology of allergic diseases and to devise low-cost diagnostics for allergic diseases.

The World Wide Web has given us an increasingly valuable tool to communicate on a regular basis with allergists around the world and to provide information and resources to anyone with an interest in allergy. Our Web site http://www.worldallergy.org provides a wealth of freely available information and educational materials, including slide sets on major allergy topics, and Continuing Medical Education programs. The *WAO Journal *is our most recent innovation, and we hope you are enjoying your complimentary issues.

Facilitating WAO in these tasks, we have the resources of the world's leading allergy experts as volunteer members of the WAO Board of Directors and the WAO Councils and Committees.

I hope I have convinced you of the need for a global allergy organization, which is also your organization!

G. Walter Canonica

President

World Allergy Organization

